# Does cancer strive to minimize the cost of gene expression?

**DOI:** 10.18632/oncotarget.22657

**Published:** 2018-06-15

**Authors:** Dvir Schirman, Idan Frumkin, Yitzhak Pilpel

**Affiliations:** Department of Molecular Genetics, Weizmann Institute of Science, Rehovot, Israel

**Keywords:** gene expression, expression cost, clonal evolution, tumor development, optimality

Growing bacteria devote ~30% of their energy and resources to gene expression, mostly transcription and translation [1]. Can cells reduce such costs, while still maintaining desired expression levels? Molecular means that reduce expression costs per protein molecule enable cells, on the one hand, to realize the necessary cellular concentrations of each protein, and on the other hand, to devote resources to other life processes. Hence, how cells can achieve a desirable expression level of a given gene while minimizing its expression costs - remains a fundamental question. We have recently demonstrated how bacterial cells have evolved means to optimize expression costs, and hypothesize here that cancer cells may also evolve in a similar manner.

Can cells evolve to minimize costs of gene expression without compromising expression levels [2]. We explored this question by measuring fitness of ~14,000 *E. coli* variants, each expressing a version of a reporter protein [2]. Since this protein serves no beneficial function, its expression only reduces cellular fitness. Each variant expressed the reporter at a different level, and higher expression resulted in lower fitness. Yet, when we normalized each variant’s fitness to the reporter’s expression level, we found that certain variants display better fitness than others that have same expression level. We designated gene architectures as ‘efficient’ if the fitness of the strains that carry them was higher than expected given their expression. We uncovered elements that minimize the cost of gene expression: (i) lowering mRNA levels, accompanied by fast translation initiation, thus reducing transcription costs; (ii) attenuating ribosomes during early elongation, for better allocation of ribosomes; (iii) using amino-acids that are less hydrophobic, as these presumably reduce aggregates formation; and (iv) using amino acids that are cheap to synthesize.

We constructed a model that predicts the ability of a sequence to minimize expression cost, and applied it to the genomes of, *E.coli* and *B.subtilis*. Notably, we observed that highly expressed genes in these genomes evolved to reduce expression costs, employing the above mechanisms.

In our study, we utilized bacteria for studying principles of cellular fitness. However, expression costs might be universal as they derive from conserved machineries. In all organism, minimizing costs while maintaining high levels would be beneficial.

While mammalian cells are not selected for rapid growth, tumors are [3]. From an evolutionary point of view, tumors constitute large genetically heterogeneous population[4, 5, 6]. Principles of neo-Darwinian evolution are manifested during the development of a tumor: clones of tumor cells expand and compete, genetic diversity arises following mutations, adaptive sub-clones are selected, and proliferative cells prevail. Cancers thus resemble evolution of bacteria due to their asexual nature, and the competition on resources.

**Figure 1:**
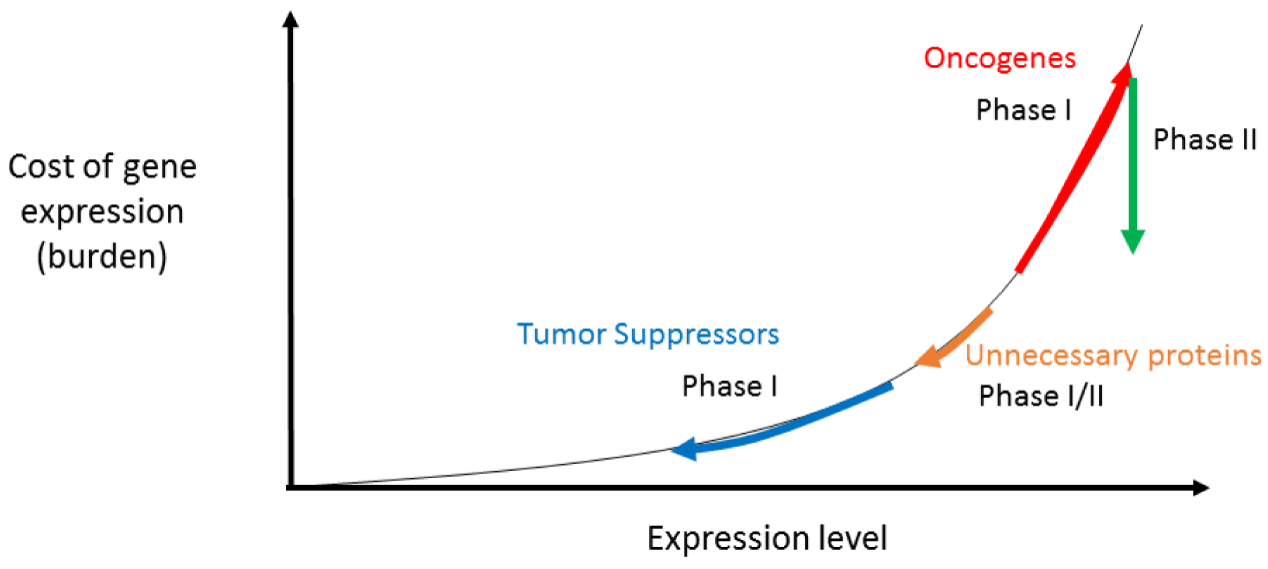
Primary and secondary adaptation of gene expression in cancer Cancer typically lowers expression levels of tumor suppressors, and increases oncogene expression. We speculate that the evolution of oncogenes within a tumor might be bi-phasic. At first, selection favors their increased expression. Then, during later stages of tumor evolution, the tumor might find molecular means to reduce the cost of gene expression, while maintaining the same expression levels (see text). tumor suppressors are selected for their reduced expression, which is naturally accompanied by reduction of costs. Furthermore, shutting down the expression of unnessary proteins, which may occur during both phases of adaptation, could also lower the overall burden on the cellular machineries. This hypothesis calls for reanalysis of cancer mutation data across tumor evolution timelines, as it would predict that mutations appearing at later stages of tumor development might lead to refinement of costs during Phase II.

We speculate that minimizing the cost of expression might serve as an adaptive mechanism in cancer. Undoubtedly, the most prominent driving forces in cancer development relate to cell cycle regulation, and evasion from immune responses. Nevertheless, vast abundance of mutations in some cancer [7] suggests that even after evolving elevated expression levels of oncogenes, “second-order adaptations” might appear that could optimize their expression costs (Figure 1).

How could cancer minimize expression costs? First, it can accumulate mutations in genes that are not needed, and reduce their expression levels. A more challenging adaptation could be that tumors act to reduce expression costs of necessary genes by adapting efficient genetic architectures.

We suggest to re-analyze cancerous mutations since some mechanisms we identified in bacteria could act in cancers. For example, costly-to-synthesize amino acids might be replaced with chemically similar, cheaper ones. Attenuating ribosomes at early elongation could be achieved by reducing tRNAs common in ORFs’ starts. Cancerous processes might reduce the levels of aggregation-prone proteins, or upregulate chaperones for protein folding.

To test such hypotheses, one could build on previous studies of bacteria, and measure the fitness of thousands of gene architectures in cell cultures. Such studies could provide further insights into the economy of cancerous tumors, identify more subtle driving mutations, and might lead to avenues for fighting cancer, by attacking its unique cellular economics.
